# Allopurinol non-covalently facilitates binding of unconventional peptides to HLA-B*58:01

**DOI:** 10.1038/s41598-023-36293-z

**Published:** 2023-06-09

**Authors:** Xuelu Huan, Nicole Zhuo, Haur Yueh Lee, Ee Chee Ren

**Affiliations:** 1 Singapore Immunology Network (SigN), Agency for Science, Technology and Research (A*STAR), 8A Biomedical Grove, Immunos, Singapore, 138648 Singapore; 2 Allergy Center and Department of Dermatology, Singapore General Hospital, Singapore, 169608 Singapore; 3 Department of Microbiology & Immunology, Yong Loo Lin School of Medicine, National University of Singapore, Singapore, 117545 Singapore

**Keywords:** X-ray crystallography, Allergy

## Abstract

Allopurinol, widely used in gout treatment, is the most common cause of severe cutaneous adverse drug reactions. The risk of developing such life-threatening reactions is increased particularly for HLA-B*58:01 positive individuals. However the mechanism of action between allopurinol and HLA remains unknown. We demonstrate here that a Lamin A/C peptide KAGQVVTI which is unable to bind HLA-B*58:01 on its own, is enabled to form a stable peptide-HLA complex only in the presence of allopurinol. Crystal structure analysis reveal that allopurinol non-covalently facilitated KAGQVVTI to adopt an unusual binding conformation, whereby the C-terminal isoleucine does not engage as a PΩ that typically fit deeply in the binding F-pocket. A similar observation, though to a lesser degree was seen with oxypurinol. Presentation of unconventional peptides by HLA-B*58:01 aided by allopurinol contributes to our fundamental understanding of drug-HLA interactions. The binding of peptides from endogenously available proteins such as self-protein lamin A/C and viral protein EBNA3B suggest that aberrant loading of unconventional peptides in the presence of allopurinol or oxypurinol may be able to trigger anti-self reactions that can lead to Stevens-Johnson syndrome/toxic epidermal necrolysis (SJS/TEN) and Drug Reaction with Eosinophilia and Systemic Symptoms (DRESS).

## Introduction

Drug induced hypersensitivity is a serious healthcare problem. Stevens-Johnson syndrome/toxic epidermal necrolysis (SJS/TEN) and Drug Reaction with Eosinophilia and Systemic Symptoms (DRESS) are severe reactions which can be fatal^1^. Allopurinol is the most common cause of such severe drug hypersensitivity^2–4^ and the majority of patients who develop SJS/TEN or DRESS are found to carry the human leukocyte antigen (HLA)-B*58:01 allele^5,6^. Existing models of drug hypersensitivity include the pharmacological-interaction (P-I) concept whereby drugs bind reversibly, and non-covalently to immune receptors such as HLA and T cell receptor^7^ as well as the altered peptide repertoire model which explains the interaction between abacavir with HLA-B*5701^8^. Previous studies showed that HLA-B*5701 is necessary for the development of abacavir hypersensitivity syndrome (AHS)^9^. Abacavir is able to alter the self-peptide loading into HLA-B*57:01, co-occupy the groove and selectively affect the formation of HLA-B*57:01-peptide complexes thus provoking immune responses^7,10–12^. Cytotoxic CD8+ T cells drove systemic reactions to abacavir by drug-specific activation which could only be mediated by HLA-B*57:01 and not closely related, non-AHS associated allotypes^13,14^. Another HLA allele, HLA-B*15:02, was strongly associated with carbamazepine (CBZ) induced SJS/TEN in the Han Chinese population, and Thai and Indian populations^15^. Despite the known pharmacogenetic association, the precise mode of interaction between allopurinol and HLA-B*58:01 remains unknown. Allopurinol is a purine analog with aromatic ring structures that closely resemble that of tryptophan^16,17^ (Supplementary Fig. 1a,b). We hypothesized that the drug may be able to replace tryptophan as the PΩ residue within the peptide binding F-pocket of the HLA-B*58:01 molecule. To test this possibility, we selected a known peptide KAGQVVTIW that has been identified by mass spectrometry analysis of HLA Class-I peptide pools eluted from purified HLA-B*58:01 of a 721.221 cell line^18^. The endogenously bound HLA-B*58:01 peptide is from a self-protein lamin A/C, and contains the preferred anchor residues at P2 (Ala, Tyr, Ser) and P9 (Trp, Phe)^19,20^.

## Materials and methods

### Generation of stable allopurinol-peptide-HLA complexes

Expression of recombinant human HLA class I allele (exon 2–4) and human β2-microglobulin (β2m) were carried out as previously described^21,22^. Briefly, soluble HLA-B*58:01 (residues 1–277) and full length β2-microglobulin (residues 1–99) with pET30a vectors were expressed in *Escherichia coli* as inclusion bodies using the pET prokaryotic expression system (Novagen Inc.). They were refolded with four peptides separately: KAGQVVTIW, VSFIEFVGW, KAGQVVTI and VSFIEFVI. The native 9mer peptides were refolded in the absence of drugs, while the truncated 8mer peptide were refolded in the presence of allopurinol or oxypurinol separately. The refolded HLA-B*58:01-peptide (pHLA) complexes were dialyzed in 10 mM Tris–HCl (pH 8.0) buffer at 4 °C overnight. They were then purified by anion exchange chromatography using HiPrep DEAE 16/10 column (GE Healthcare), and pHLA complexes were eluted with buffer containing 10 mM Tris–HCl, pH 8.0 and 1 M NaCl. The HiLoad 16/600 Superdex 75 preparatory-grade column (GE Healthcare) was used to further purify pHLA complexes by size exclusion chromatography and the pHLA complexes were eluted using buffer 10 mM Tris–HCl, pH 8.0. Samples of the elution peaks were loaded onto native PAGE gel and transferred onto PVDF blotting membrane and the full length gel lane strips were probed with W6/32 mouse monoclonal antibody as primary antibody followed by HRP-conjugated goat anti-mouse antibody as secondary antibody. Finally, Vivaspin centrifugal concentrators (Sartorius) were used to combine and concentrate the correctly refolded peaks corresponding to pHLA complexes to 10 mg/ml.

### Thermal stability assay of allopurinol-peptide-HLA complexes

The stability properties of the HLA-B*58:01-peptide complexes were further investigated by thermal stability assay with both 9mer peptides (KAGQVVTIW, VSFIEFVGW) and 8mer peptides (KAGQVVTI, VSFIEFVI) as described previously^7,23^. Two different protein batches of HLA-B*58:01-peptide complex were tested by using a real time PCR system LightCycler II 480 (Roche), with temperature increased from 20 to 95 °C with continuous ramp rate of 0.04 °C/s with 15 acquisitions/°C. The unfolding was monitored by using fluorescent dye Sypro orange (Molecular Probes), with the fluorescence intensity measured with excitation at 490 nm and emission at 575 nm. Each of the samples were set in triplicates at a concentration of 20 µM in 10 mM Tris–HCl buffer, pH 8.0. The data plotted as negative first derivatives of fluorescence change against temperature, whereby, the melting temperature (Tm) is defined as the minima in the negative peak (-dRFU/dT).

### Protein crystallization

Commercially available crystallization kits from Hampton Research were used to initiate the crystal screening for purified pHLA complexes. By employing Phoenix protein crystallization robot (Art Robbins Instruments), the pHLA complexes and crystallization solution drops were dispensed in equal volumes (1:1) into 96 well plates (Art Robbins Instruments). After setting crystal screening, plates were incubated at 20 °C. Once the initial hits were obtained, the crystal drops for pHLA complexes were set using hanging drop vapor diffusion method in 24 well plates (Hampton Research) and plates were incubated at 20 °C. The crystals for HLA-B*58:01-KAGQVVTIW in the absence of allopurinol and oxypurinol, and HLA-B*58:01-KAGQVVTI complex in the presence of 10 µg/ml allopurinol formed in condition containing 0.2 M sodium thiocyanate pH 6.9, 20% w/v polyethylene glycol 3350. Protein crystals of HLA-B*58:01-VSFIEFVGW in the absence of allopurinol and oxypurinol, and HLA-B*58:01-VSFIEFVI in the presence of 10 µg/ml allopurinol formed in condition containing 0.04 M citric acid, 0.06 M bis–Tris propane pH 6.4, 20% w/v polyethylene glycol 3350. The pHLA complex crystals were cryo-protected by soaking in a reservoir solution supplemented with 20% glycerol, harvested by different sized CryoLoops (Hampton Research) and stored in liquid nitrogen before use.

### X-ray diffraction data, processing and structure determination

The TPS-05A beamline and the CCD detector MX300-HS was used at the Taiwan Photon Source of National Synchrotron Radiation Research Centre to collect the X-ray diffraction data. HKL2000 suite^24^ and XDS programs^25^ were used to index, integrate and scale diffraction data. All structures were solved by molecular replacement using Phaser^26^ and the previously determined HLA-B*:5801 molecule used as a search model (PDB code: 5IM7)^27^ with peptide and water molecules removed. The model building was performed manually with program COOT^28^ and the refinement was carried out using Refamc5^29^ and Phenix^30^. The quality of the final model was validated using PROCHECK^31^ and figures were prepared with PyMol^32^. The atomic coordinates and structure factors have been deposited in the Protein Data Bank (PDB). Accession code for HLA-B*58:01-KAGQVVTIW is 7WZZ; HLA-B*58:01-KAGQVVTI is 7X1B; HLA-B*58:01-VSFIEFVGW is 7X00; HLA-B*58:01-VSFIEFVI is 7X1C (Table 1).

**Table 1:** Data collection and refinement statistics.

Parameter	Value(s) for^a^			
	HLA-B*58:01-KAGQVVTIW (7WZZ)	HLA-B*58:01-KAGQVVTI (7X1B)	HLA-B*58:01-VSFIEFVGW (7X00)	HLA-B*58:01-VSFIEFVI (7X1C)
Data collection				
Space group	P2_1_2_1_2_1_	P2_1_2_1_2_1_	P2_1_2_1_2_1_	P2_1_2_1_2_1_
Unit cell dimensions (Å)				
* a*	50.77	50.44	50.72	50.72
* b*	81.58	81.72	82.06	82.16
* c*	109.43	109.23	110.28	110.08
α, β, γ (°)	90, 90, 90	90, 90, 90	90, 90, 90	90, 90, 90
Resolution (Å)	50–1.31 (1.35–1.31)	50–1.40 (1.45–1.40)	50–1.45 (1.50–1.45)	50–1.41 (1.46–1.41)
* I*/σ*I*	23.5 (3.7)	20.0 (1.7)	14.4 (2.9)	17.64 (1.56)
CC1/2 (%)	99.9 (91.3)	99.9 (60.5)	99.8 (90.6)	99.9 (59.5)
Completeness (%)	98.5 (96.9)	98.0 (96.0)	98.4 (96.5)	99.9 (99.5)
Redundancy	7.3 (7.3)	7.3 (7.4)	7.4 (6.9)	7.2 (7.2)
Refinement				
Resolution (Å)	50–1.31	50–1.40	50–1.45	50–1.41
No. of reflections	109,358	87,950	80,874	89,414
* R*_work_/*R*_free_ (%)	14.3/16.9	15.4/17.9	14.3/16.8	15.2/18.2
Avg B factor (Å^2^)				
Protein	17.2	21.0	23.5	22.7
Peptide	17.4	18.9	21.0	19.8
Water	35.9	35.0	37.7	36.6
Glycerol	33.1	36.0	47.7	49.2
r.m.s.d.				
Bond length (Å)	0.012	0.008	0.005	0.009
Bond angle (°)	1.24	1.13	0.8	1.36
Ramachandran plot				
Favored regions (%)	98.4	98.4	98.7	98.9
Allowed regions (%)	1.6	1.4	1.3	0.9
Outlier		0.2		0.2

r. m. s. deviations: root-mean-square-deviation.

^a^Diffraction data from one crystal was merged into a complete dataset.

## Results

### HLA-B*58:01 forms a stable protein complex with human lamin A/C peptide

**Figure 1:**
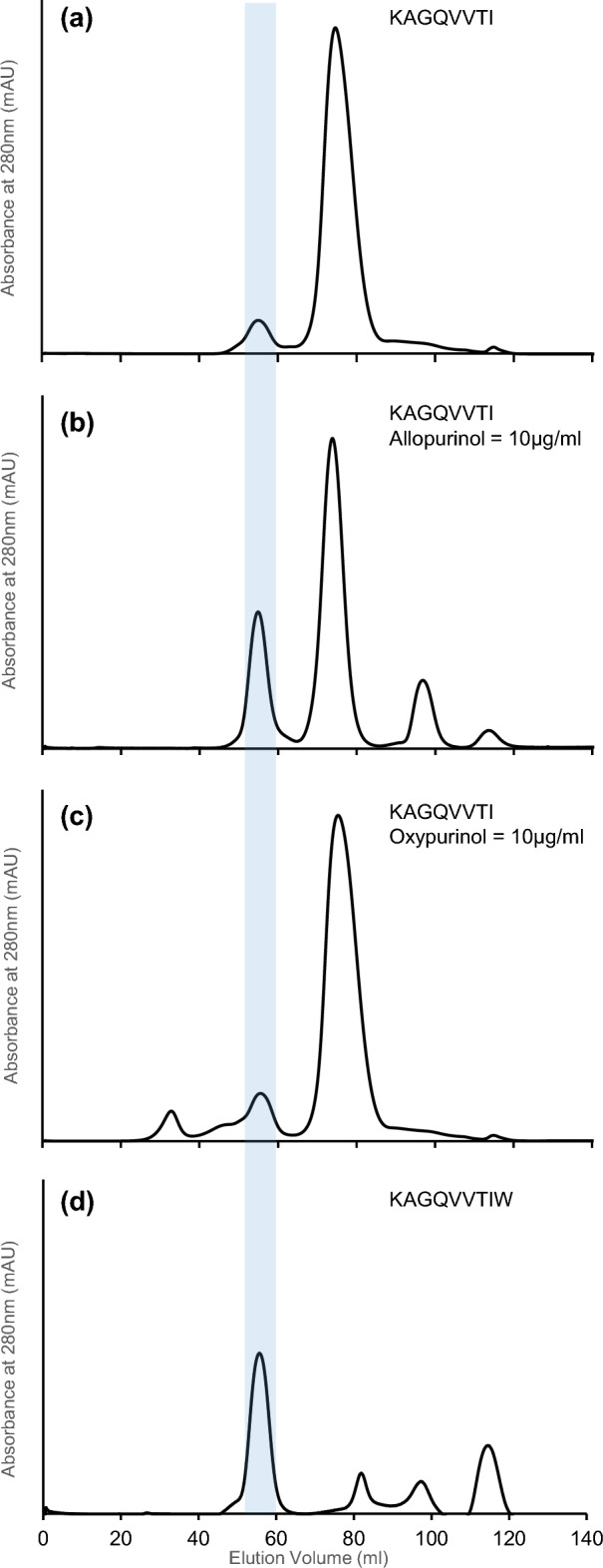
KAGQVVTI forms stable peptide-HLA-B*58:01 complex in the presence of allopurinol. Size exclusion chromatography with a HiLoad 16/600 Superdex 75 preparatory-grade GF column of HLA-B*58:01 refolded with: (**a**) KAGQVVTI without allopurinol or oxypurinol. (**b**) KAGQVVTI with 10 µg/ml allopurinol. (**c**) KAGQVVTI with 10 µg/ml oxypurinol. (**d**) Native 9mer KAGQVVTIW without allopurinol or oxypurinol, was used as a positive control and elution position marker. The correctly refolded peptide-HLA complex (**d**) elutes between 50 and 60 ml of elution volume and is marked by a vertical light blue bar for easy reference.

KAGQVVTIW, the native 9mer fragment from human lamin A/C was synthesized as well as a truncated 8mer KAGQVVTI without the C-terminal “W”. The KAGQVVTIW peptide was mixed with HLA-B*58:01 heavy chain and β2-microglobulin (β2m) in a refolding buffer^21,22^ and the resulting peptide-HLA complex was successfully formed and can be detected as an elution peak between 50 and 60 ml elution volume using a FPLC Superdex-75 column (Fig. 1d). Without the C-terminal tryptophan, the KAGQVVTI peptide failed to form peptide-HLA complex efficiently and only a minimal amount of product was detected (Fig. 1a). On addition of allopurinol (10 µg/ml) to the KAGQVVTI refolding mix, the capacity to form peptide-HLA complex was restored as shown by the strong peak, corresponding to that of the positive control 9mer peptide (Fig. 1b). Allopurinol has plasma half-life of 1–2 h^33^, and is rapidly metabolized in the liver to produce oxypurinol, a pharmacologic active inhibitor of xanthine oxidase with a longer plasma-half-life of 23 h^34^. As oxypurinol is closely related in structure to allopurinol and has a longer plasma half-life (Supplementary Fig. 1c), it may facilitate peptide loading in a similar function in a similar manner as allopurinol. When the same refolding assay above was performed using oxypurinol, the results in Fig. 1c shows that oxypurinol appears to be less effective than allopurinol in assisting peptide-HLA formation. The peptide-HLA-B*58:01 complexes purified by size exclusion chromatography were then investigated by western blot analysis (Supplementary Fig. 2). Samples of the elution peaks were loaded onto native PAGE gel and transferred onto PVDF blotting membrane. W6/32 mouse monoclonal antibody was used as primary antibody, HRP-conjugated goat anti-mouse antibody was used as secondary antibody. Native HLA-B*58:01-KAGQVVTIW complex sample without allopurinol or oxypurinol, was used as a positive control. The results showed that the HLA-B*58:01-KAGQVVTI in presence of either allopurinol or oxypurinol refolded well as 9mer peptide-HLA complex.

**Figure 2:**
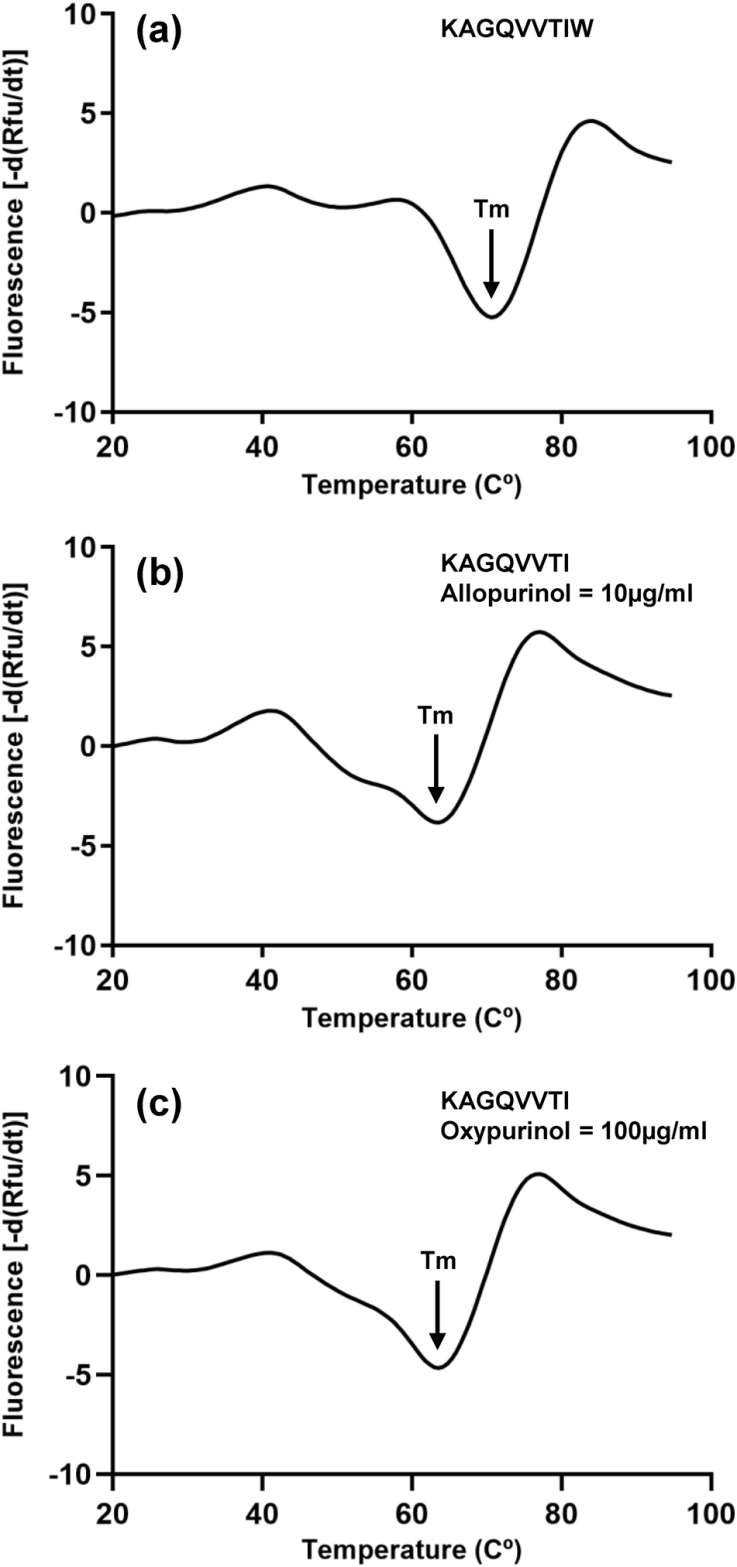
Thermal stability of the HLA-B*58:01-peptide complexes. The Tm of (**a**) HLA-B*58:01-KAGQVVTIW, (**b**) HLA-B*58:01-KAGQVVTI with 10 µg/ml allopurinol, and (**c**) HLA-B*58:01-KAGQVVTI with 100 µg/ml oxypurinol was 71 °C, 65 °C and 65 °C, respectively.

The stability of the HLA-B*58:01-peptide complexes were further investigated by thermal stability assay with both 9mer peptide (KAGQVVTIW) and 8mer peptide (KAGQVVTI). Two different protein batches of HLA-B*58:01-peptide complex were tested, their unfolding was monitored by using fluorescent dye Sypro orange. The data showed the average melting temperature (Tm) is 71 °C for HLA-B*58:01-KAGQVVTIW (Fig. 2a) and 65 °C for allopurinol-KAGQVVTI-HLA-B*58:01 (Fig. 2b) and oxypurinol-KAGQVVTI-HLA-B*58:01 (Fig. 2c). The relatively high thermal stability of the 8mers achieved only in the presence of allopurinol or oxypurinol suggest that the drugs have enabled the loading of sub-optimal peptides that normally would not be able to properly bind HLA-B*58:01.

### HLA-B*58:01 form stable protein complex with EBV peptide

Next, we asked the question whether the capacity of allopurinol to facilitate binding of unconventional peptides to HLA-B*58:01 can be repeated using a second peptide. To this end, we used VSFIEFVGW, a 9mer from the Epstein-Barr Virus (EBV) EBNA3B protein, which is known to be restricted by HLA-B*58:01 in a CTL cytotoxicity assay^35^. The 8mer VSFIEFVG was also synthesized, together with an 8mer VSFIEFVI with isoleucine as PΩ, so as to be consistent with the KAGQVVTI peptide PΩ residue. However, VSFIEFVG does not form peptide-HLA complex neither in the absence or presence of allopurinol and oxypurinol (Supplementary Fig. 3a). In contrast the VSFIEFVI peptide readily formed successful complex with HLA-B*58:01 (Supplementary Fig. 3d) with 10 µg/ml allopurinol. With this empirical outcome, VSFIEFVI was used for further analysis since the P8-Ile is consistent with both KAGQVVTI and VSFIEFVI peptides.

When refolded with HLA-B*58:01, the 8mer was unable to form distinct peptide-HLA complex (Supplementary Fig. 3b). Similar to what was observed with the KAGQVVTI (Fig. 1b), the addition of allopurinol dramatically improved the generation of peptide-HLA product (Supplementary Fig. 3d). The P2-Ser of VSFIEFVI being a favored anchor residue was able to function like P2-Ala of KAGQVVTI in providing stability at the N-terminal part of the peptide, and this facilitated the other peptide residues to engage and form bonding interactions with the HLA-B*58:01 molecule. VSFIEFVI in the presence of added oxypurinol (100 µg/ml) was able to produce a small amount of refolded product (Supplementary Fig. 3e).

The stability properties of the HLA-B*58:01-VSFIEFVGW and HLA-B*58:01-VSFIEFVI complexes were also investigated by using fluorescent dye Sypro orange to perform the thermal stability assay. Two different protein batches of HLA-B*58:01-peptide complex were tested, the data showed the average melting temperature (Tm) is 70 °C for HLA-B*58:01-VSFIEFVGW (Supplementary Fig. 4a) and 57 °C for both HLA-B*58:01-VSFIEFVI with allopurinol (Supplementary Fig. 4b), or with oxypurinol (Supplementary Fig. 4c). This data demonstrate that HLA-B*58:01-peptide complex become much more stable with the help of allopurinol or oxypurinol. Taken together, this data demonstrates that allopurinol, and to a lesser extent oxypurinol, may enable the loading of unconventional peptides that are normally suboptimal in forming stable peptide-HLA-B*58:01 complexes.

### HLA-B*58:01 protein crystal structural analysis with different peptides

**Figure 3:**
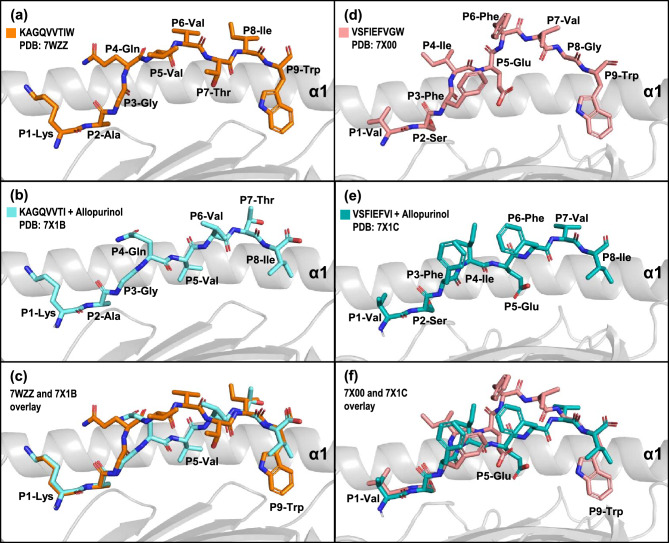
Unconventional peptides use only one peptide binding pocket. Structural comparison of peptides in the binding groove of HLA-B*58:01. The main chain of HLA-B*58:01 is depicted schematically as cartoon (light grey) and for clarity only helix-1 (α1) is shown and the peptides are represented in stick. (**a**) KAGQVVTIW (in orange, PDB: 7WZZ) and (**d**) VSFIEFVGW (in light pink, PDB: 7X00) adopted bulged conformation in the middle portion of the peptide. The 8mer peptides (**b**) KAGQVVTI (cyan) and (**e**) VSFIEFVI (teal) formed protein crystals with HLA-B*58:01 in the presence of 10 µg/ml allopurinol separately. The peptides occupied the binding groove and displayed an extended conformation with the side-chain of P5 oriented downwards. (**c**,**f**) Overlay of 7WZZ vs 7X1B, and 7X00 vs 7X1C respectively clearly illustrates the downward orientation of the middle portion (P4–P6) of the short peptides that enables additional bond interactions with the HLA-B*58:01 molecule. The PΩ isoleucine in (**b**) and (**e**) are seen to align in the exact position as the P8 of the longer 9mers and do not extend into the F-pocket. Allopurinol does not appear in these crystals as it associates only non-covalently, it is likely to have moved during the process of crystal packing.

The refolding experiments above do not fully explain how KAGQVVTI and VSFIEFVI being 8mers achieve stable binding, and whether the P8-isoleucine function as the PΩ residue and thereby fit into the F-pocket of HLA-B*58:01. To clarify this, we generated protein crystals of allopurinol-KAGQVVTI-B*58:01 (PDB: 7X1B) and allopurinol-VSFIEFVI-B*58:01 (PDB: 7X1C). The corresponding native 9mer without allopurinol were also generated: KAGQVVTIW-B*58:01 (PDB: 7WZZ) and VSFIEFVGW-B*58:01 (PDB: 7X00)—these will function as the reference to compare the relative orientations of the truncated 8mer vs native 9mer peptides. The solved structures of 7WZZ (Fig. 3a) and 7X00 (Fig. 3d) show both 9mer peptides to adopt a slightly bulged conformation with the middle P4-P6 residues oriented upwards towards the direction of the T cell receptor. The PΩ-Trp residue of both peptides occupy their respective F-pocket of HLA-B*58:01, which is typical of canonical peptide binding behavior. While an 8mer is of sufficient length to occupy both binding pockets of the peptide binding groove, only P2-Ala of KAGQVVTI (Fig. 3b) and P2-Ser VSFIEFVI (Fig. 3e) engages the binding pocket. This is clearly observed in the overlay of the 8mer with their corresponding native 9mer peptides (Fig. 3c,f). Importantly, the isoleucine of KAGQVVTI and VSFIEFVI do not engage the F-pocket to take the place of the missing tryptophan. As KAGQVVTI uses P2-Ala and VSFIEFVI uses P2-Ser as the only anchor residues, it makes the peptides unconventional in terms of binding capacity and this structural data confirm and explain the lack of a peptide-HLA peak in Fig. 1a and Supplementary Fig. 3b. The binding capacity of these 2 unconventional peptides can be significantly enhanced by the addition of allopurinol, which closely resembles the molecular size and structure of tryptophan. Allopurinol therefore facilitates the binding of the two unconventional peptides to generate peptide-B*58:01 complexes that are sufficiently stable to allow protein crystals be made. Allopurinol, acting as a tryptophan substitute likely assists in stabilizing the initial formation of the peptide-HLA complex and its residency may only be transient since the drug is in free form and therefore acts non-covalently. This could explain why the drug does not appear in Fig. 3b,e. Functionally, even if present in the binding F-pocket, allopurinol or oxypurinol do not make direct contact with the T cell receptor as it is buried.

**Figure 4:**
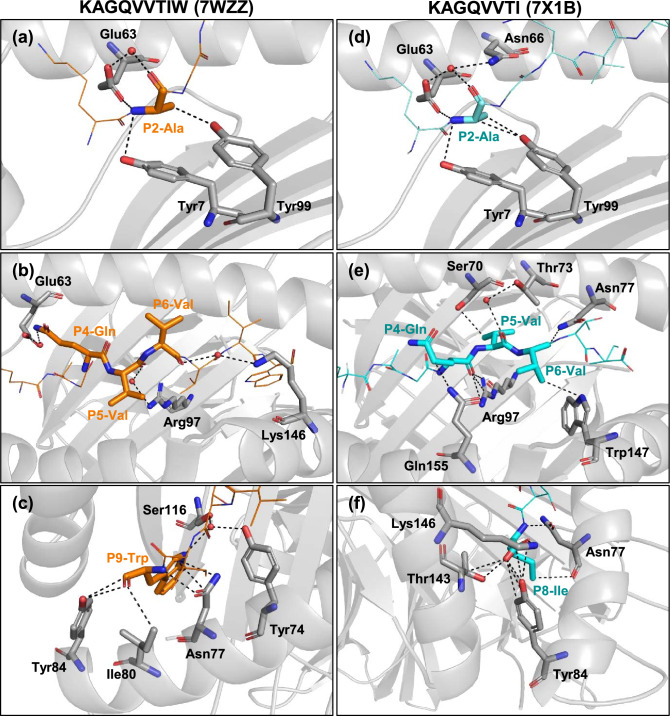
Hydrogen bond interactions occurring in allopurinol-peptide-HLA stabilized complex. The main chain of HLA-B*58:01 is depicted schematically in light grey with selected residues shown as stick. Left panel shows interactions for KAGQVVTIW (in orange, PDB: 7WZZ), right panel for KAGQVVTI (in cyan, PDB: 7X1B). (**a**) shows interactions between P2-Ala of KAGQVVTIW with HLA-B*58:01. (**b**) shows interactions between P4-Gln, P5-Val and P6-Val of KAGQVVTIW with HLA-B*58:01. (**c**) shows interactions between P9-Trp of KAGQVVTIW with HLA-B*58:01. Similarly, panel (**d**) shows interactions between P2-Ala of KAGQVVTI with HLA-B*58:01, (**e**) shows interactions between P4-Gln, P5-Val and P6-Val of KAGQVVTI with HLA-B*58:01. (**f**) shows interactions between P8-Ile of KAGQVVTI with HLA-B*58:01. Hydrogen bonding is indicated as black dotted line, and peptides are displayed as stick.

The lack of a covalently linked PΩ tryptophan allowed KAGQVVTI and VSFIEFVI to adopt a different orientation compared to their counterpart residues in the 9mer peptides. As the 8mer peptides are more stretched instead of bulging upwards, it has opportunity to establish additional bonding interactions with the HLA molecule that would account for its stability. It can be seen that the P2-Ala of KAGQVVTIW (7WZZ) form direct hydrogen bonds with Glu63 on α1 helix and Tyr7, Tyr99 on β sheets of the pocket B of HLA-B*58:01 (Fig. 4a). The P2-Ala of KAGQVVTI (7X1B) forms one more hydrogen bond with Tyr99 compared to the P2-Ala of 9mer peptide (Fig. 4d). Consistent with previous studies, the peptide residues interacted with HLA heavy chain via water molecules, showing that major histocompatibility complex (MHC)–peptide binding stability is assisted by bound water molecules^36^. P2-Ala of KAGQVVTI (7X1B) has hydrogen bond connections with Glu63 and Asn66 via one water molecule (Fig. 4d), while P2-Ala of KAGQVVTIW (7WZZ) only make water mediated hydrogen bond connections with Glu63 (Fig. 4a). At the C-terminal end of the peptides, P9-Trp on KAGQVVTIW (7WZZ) forms direct hydrogen bonds with Asn77, Ile80 and Tyr84 on α1 helix (Fig. 4c). The extended conformation of KAGQVVTI (7X1B) makes the main P8-Ile shift towards α2 helix, and forms direct hydrogen bonds with Asn77, Tyr84 on α1 helix and Thr143, Lys146 on α2 helix (Fig. 4f), which also contribute to its stability. The indole ring of P9-Trp on KAGQVVTIW (7WZZ) interacts with Asn77 on α1 helix directly, it also has water mediated hydrogen bond interactions with Tyr74 on α1 helix and Ser116 on β sheet via one water molecule. Compared to this, the side chain of P8-Ile on KAGQVVTI (7X1B) forms a hydrogen bond with Asn77 on α1 helix. The most notable difference in binding interactions between KAGQVVTIW and KAGQVVTI occur in the middle part of peptides, where the P4, P5 and P6 residues help to support the protein-peptide complex stability by means of secondary anchor bonding. With HLA-B*58:01-KAGQVVTIW (7WZZ) due to the bulged conformation P4-Gln, P5-Val and P6-Val have no direct hydrogen bond interactions with the HLA-B*58:01 molecule (Fig. 4b). The only interactions among this region are P4-Gln with Glu63, P5-Val with Arg97, and P6-Val with Lys146 mediated via three water molecules separately. In contrast, the extended confirmation of 8mer KAGQVVTI peptide causes the middle part of peptide to be buried deeper in the peptide binding groove. The P4-Gln residue forms hydrogen bonds with Arg97 on β sheet and Gln155 on α2 helix (Fig. 4e). P5-Val interacts with Ser70 on α1 helix. P6-Val forms hydrogen bonds with Asn77 on α1 helix and Trp147 on α2 helix. Moreover, the P5-Val residue also interacts with Thr73 on α1 helix via one water molecule. Therefore the re-oriented conformation of the KAGQVVTI peptide enables additional hydrogen bond interactions at P4-P6 that stabilized an otherwise unconventional binding without a PΩ anchor.

This pattern of a network of hydrogen bonds extending across almost the entire length of the peptide from P2-P8 and interacting with the HLA-B*58:01 molecule can be seen in a second peptide (7X1C, Fig. 3e). The P2-Ser of 7X00 and 7X1C form direct hydrogen bonds with Glu63 and Asn66 on α1 helix, and Tyr99 on β sheet (Supplementary Fig. 5a,d). The side chain of P2-Ser of 7X00 forms one more hydrogen bond with Met 67 (Supplementary Fig. 5a), while P2-Ser of 7X1C forms one more hydrogen bond with Tyr7 on β sheet of the pocket B (Supplementary Fig. 5d).

The P9-Trp of 7X00 forms direct hydrogen bonds with Asn77, Tyr84 on α1 helix, and Thr143 on α2 helix (Supplementary Fig. 5c). The indole ring of P9-Trp also forms hydrogen bond with Asn77 on α1 helix directly, and interacts with Tyr74 on α1 helix and Ser116 on β sheet via one water molecule (Supplementary Fig. 5c). Without the P9-Trp, the isoleucine of VSFIEFVI (7X1C) forms direct hydrogen bonds with Asn77, Tyr84 on α1 helix and Lys146 on α2 helix (Supplementary Fig. 5f.). The lack of the P9-Trp also allows the 8mer to reside deeper in the middle of the peptide binding cavity where the P4-Ile of 7X1C makes hydrogen interactions with Arg97 on the β sheet (Supplementary Fig. 5e). The side chain of P5-Glu forms hydrogen bonds with Tyr74 on the α1 helix and Arg97 on the β sheet tightly. P6-Phe forms a hydrogen bond with Asn77 on the α1 helix. In contrast, the 9mer 7X00 shows the P4-P6 residues adopt a bulged conformation away from the peptide binding cavity such that there is no hydrogen bond forming between P4-Ile residue and HLA-B*58:01 molecule. The P5-Glu has only one hydrogen bond with Arg97 on the β sheet, and P6-Phe with Gln155 on the α2 helix (Supplementary Fig. 5b).

Allopurinol is metabolized to oxypurinol in 1–2 h, and as the latter has a half-life of 23 h, it has a longer dwell time in the body. We observed that oxypurinol is not as effective compared to allopurinol in facilitating the binding of unconventional peptides (Fig. 1c). By scaling up the refolding quantities, we were able to collect sufficient amount of oxypurinol-VSFIEFVI-B*58:01 monomers for crystallization experiments, however the yield for oxypurinol-KAGQVVTI-B*58:01 did not reach the required amount for crystallization. The solved structure of oxypurinol-VSFIEFVI-B*58:01 showed that the peptide adopted a similar flat orientation as its allopurinol counterpart (Supplementary Fig. 6a), and it was compared with VSFIEFVGW-B*58:01 (Supplementary Fig. 6b). The overlay of allopurinol-VSFIEFVI-B*58:01 and oxypurinol-VSFIEFVI-B*58:01 showed remarkable similarity (Supplementary Fig. 6c), suggesting that even though oxypurinol is less efficient than allopurinol in facilitating peptide binding to HLA-B*58:01, once loaded, the peptide orientation is very similar.

## Discussion

Although HLA-B*58:01 has been identified as a strong risk factor for allopurinol induced SJS/TEN, the mechanism of drug-HLA interaction is not known. Attempts to explain the interaction using the abacavir model of altered peptide binding has not been successful. The pharmacologic-interaction (p-i) model which suggest that drugs can bind non-covalently and reversibly was also considered in this study. Using 8mer peptides that have only one favorable anchor peptide residue at the N-terminal, we showed that such peptides are indeed suboptimal and do not bind to HLA-B*B58:01. Interestingly, the presence of allopurinol and to a lesser extent oxypurinol, enabled such suboptimal peptides to form stable complexes with HLA-B*58:01, with a Tm that is sufficient to allow protein crystals to be generated. This unusual stable binding is explained by the re-orientation of the P4-P6 residues downwards instead of the conventional upward bulged conformation seen in the native 9mer peptide. The re-oriented P4-P6 form secondary bond interactions that contributes to its stability.

The preferred amino acid anchor residues at the F-pocket appear to be similar for both B*57:01 and B*58:01, but the occurrence of allopurinol related SJS/TEN among B*57:01 individuals is rare, and conversely the occurrence of abacavir related hypersensitivity among B*58:01 individuals is also rare. This suggest that allopurinol does not interact the same way with B*57:01 as it does with B*58:01. It is possible that other factors such as the effect of secondary pockets along the binding groove may result in different bound peptide repertoires^37^. Future studies of the peptide repertoires of B*58:01 in the presence and absence of allopurinol should be investigated. However, it is worthwhile to note that there are B*58:01 individuals who are tolerant to allopurinol^5,38^. As the B*58:01 allele is the same in both susceptible and tolerant cohorts, it is likely that the peptide repertoires that could be recovered from B*58:01 expressing T2 cells may not necessarily distinguish SJS/TEN susceptible from tolerant phenotypes.

One limitation of this study is that the allopurinol and oxypurinol were not visible in the solved pHLA structures. The pharmacologic-interaction (p-i) model propose that drug binding is reversible and this is the likely explanation that the drugs were functional during the formation of the pHLA complex, but being non-covalent, the drugs were transiently bound at the F-pocket. In support of this, the FPLC elution data clearly demonstrates the involvement and requirement of allopurinol and oxypurinol in the formation of peptide-HLA complexes with the suboptimal 8mer peptides. The crystal structures 7X1B and 7X1C do show a flattening of the 8-mer peptide and especially at the region that would normally engage the CDR3 loop of TCR. This could possibly result in reduced contact of peptide with TCR and thus slowing the kinetics of T cell activation. At the same time, lower affinity TCRs may also be activated thereby broadening the range of T cells activated. Interestingly, without the peptide C-terminal residue being held in place deeper into the F-pocket, the P8 could also be free to flex upwards towards the TCR causing activation by “induced fit”. Thus the binding of such unconventional peptides could result in activation of T cells that would not normally occur.

In comparing with the abacavir mode of drug-HLA interaction^7^ the allopurinol-HLA-B*58:01 interaction shows similarity in that the last amino acid of the unconventional 8mer peptide occupies the shallow upper portion of the F-pocket where the binding requirements are not as restricted as that with the deeper portion normally occupied by tryptophan. This could alter and widen the repertoire of permissible unconventional peptides that bind to B*58:01 in the presence of allopurinol and oxypurinol. We have provided evidence for two such permissible peptide motifs: P1-(P2-Ala)-P3-P4-P5-P6-P7-(P8/PΩ-Ile) and P1-(P2-Ser)-P3-P4-P5-P6-P7-(P8/PΩ-Ile) that can be aberrantly bound to HLA-B*58:01, and other permissible peptide motifs likely exist. The binding of peptides from endogenously available proteins such as self-protein lamin A/C and viral protein EBNA3B suggest that aberrant loading of unconventional peptides in the presence of allopurinol or oxypurinol may be able to prime the immune system and trigger anti-self reactions that can lead to SJS/TEN and DRESS.

## Supplementary Information

Supplementary Figures.

## Data Availability

The atomic coordinates and structure factors for the reported crystal structures have been deposited in the Protein Data Bank under accession codes 7WZZ (10.2210/pdb7WZZ/pdb), 7X1B (10.2210/pdb7X1B/pdb), 7X00 (10.2210/pdb7X00/pdb) and 7X1C (10.2210/pdb7X1C/pdb).

## References

[1] Jaruthamsophon K, Thomson PJ, Sukasem C, Naisbitt DJ, Pirmohamed M (2022). HLA allele-restricted immune-mediated adverse drug reactions: Framework for genetic prediction. Annu. Rev. Pharmacol. Toxicol..

[2] Halevy S (2008). Allopurinol is the most common cause of Stevens-Johnson syndrome and toxic epidermal necrolysis in Europe and Israel. J. Am. Acad. Dermatol..

[3] Saito Y (2016). Clinical Pharmacogenetics Implementation Consortium (CPIC) guidelines for human leukocyte antigen B (HLA-B) genotype and allopurinol dosing: 2015 update. Clin. Pharmacol. Ther..

[4] Miliszewski MA, Kirchhof MG, Sikora S, Papp A, Dutz JP (2016). Stevens-Johnson syndrome and toxic epidermal necrolysis: An analysis of triggers and implications for improving prevention. Am. J. Med..

[5] Hung S-I (2005). HLA-B*5801 allele as a genetic marker for severe cutaneous adverse reactions caused by allopurinol. Proc. Natl. Acad. Sci..

[6] Wu R (2016). Impact of HLA-B*58:01 allele and allopurinol-induced cutaneous adverse drug reactions: Evidence from 21 pharmacogenetic studies. Oncotarget.

[7] Illing PT (2012). Immune self-reactivity triggered by drug-modified HLA-peptide repertoire. Nature.

[8] Pichler WJ (2019). Immune pathomechanism and classification of drug hypersensitivity. Allergy.

[9] Martin AM (2004). Predisposition to abacavir hypersensitivity conferred by HLA-B*5701 and a haplotypic Hsp70-Hom variant. Proc. Natl. Acad. Sci. USA.

[10] Ostrov DA (2012). Drug hypersensitivity caused by alteration of the MHC-presented self-peptide repertoire. Proc. Natl. Acad. Sci. USA.

[11] Norcross MA (2012). Abacavir induces loading of novel self-peptides into HLA-B*57: 01: An autoimmune model for HLA-associated drug hypersensitivity. AIDS.

[12] Illing PT (2021). Kinetics of abacavir-induced remodelling of the major histocompatibility complex class I peptide repertoire. Front. Immunol..

[13] Illing PT, Vivian JP, Purcell AW, Rossjohn J, McCluskey J (2013). Human leukocyte antigen-associated drug hypersensitivity. Curr. Opin. Immunol..

[14] Chessman D (2008). Human leukocyte antigen class I-restricted activation of CD8+ T cells provides the immunogenetic basis of a systemic drug hypersensitivity. Immunity.

[15] Chung WH (2004). A marker for Stevens–Johnson syndrome. Nature.

[16] Robins RK (1956). Potential purine antagonists. I. Synthesis of some 4,6-substituted pyrazolo [3,4-d] pyrimidines 1. J. Am. Chem. Soc..

[17] Elion GB (1989). The purine path to chemotherapy. Science.

[18] Barber LD (1997). Polymorphism in the α1 helix of the HLA-B heavy chain can have an overriding influence on peptide-binding specificity. J. Immunol..

[19] Vita R (2019). The Immune Epitope Database (IEDB): 2018 update. Nucleic Acids Res..

[20] O’Donnell TJ (2018). MHCflurry: Open-source class I MHC binding affinity prediction. Cell Syst..

[21] Garboczi DN, Hung DT, Wiley DC (1992). HLA-A2-peptide complexes: Refolding and crystallization of molecules expressed in Escherichia coli and complexed with single antigenic peptides. Proc. Natl. Acad. Sci..

[22] Liu J, Chen KY, Ren EC (2011). Structural insights into the binding of hepatitis B virus core peptide to HLA-A2 alleles: Towards designing better vaccines. Eur. J. Immunol..

[23] Layton CJ, Hellinga HW (2011). Quantitation of protein-protein interactions by thermal stability shift analysis. Protein Sci..

[24] Otwinowski Z, Minor W (1997). Processing of X-ray diffraction data collected in oscillation mode. Methods Enzymol..

[25] Kabsch W (2010). XDS. Acta Crystallogr. D Biol. Crystallogr..

[26] McCoy AJ (2007). Phaser crystallographic software. J. Appl. Crystallogr..

[27] Li X (2016). Crystal structure of HLA-B*5801, a protective HLA allele for HIV-1 infection. Protein Cell.

[28] Emsley P, Lohkamp B, Scott WG, Cowtan K (2010). Features and development of Coot. Acta Crystallogr. D Biol. Crystallogr..

[29] Murshudov GN (2011). REFMAC 5 for the refinement of macromolecular crystal structures. Acta Crystallogr. D Biol. Crystallogr..

[30] Liebschner D (2019). Macromolecular structure determination using X-rays, neutrons and electrons: Recent developments in Phenix. Acta Crystallogr. D Struct. Biol..

[31] Laskowski RA, MacArthur MW, Moss DS, Thornton JM (1993). PROCHECK: A program to check the stereochemical quality of protein structures. J. Appl. Crystallogr..

[32] Schrödinger, L. L. C. *The PyMol Molecular Graphics System*, Versión 1.8. (2015).

[33] Murrell GAC, Rapeport WG (1986). Clinical pharmacokinetics of allopurinol. Clin. Pharmacokinet..

[34] Day RO (2007). Clinical pharmacokinetics and pharmacodynamics of allopurinol and oxypurinol. Clin. Pharmacokinet..

[35] Lee SP (2000). CTL control of EBV in nasopharyngeal carcinoma (NPC): EBV-specific CTL responses in the blood and tumors of NPC patients and the antigen-processing function of the tumor cells. J. Immunol..

[36] Petrone PM, Garcia AE (2004). MHC-peptide binding is assisted by bound water molecules. J. Mol. Biol..

[37] Gfeller D (2018). The length distribution and multiple specificity of naturally presented HLA-I ligands. J. Immunol..

[38] Chiu MLS (2012). Association between HLA-B*58:01 allele and severe cutaneous adverse reactions with allopurinol in Han Chinese in Hong Kong. Br. J. Dermatol..

