# Supplementary material for: Allopurinol non-covalently facilitates binding of unconventional peptides to HLA-B*58:01
Source: Sci Rep. 2023 Jun 9;13:9373. doi: 10.1038/s41598-023-36293-z (PMC10256732; doi:10.1038/s41598-023-36293-z)
Supplement: Supplementary file 1 — Supplementary Figures. [file 41598_2023_36293_MOESM1_ESM.pdf]

Allopurinol Non-Covalently Facilitates Binding of Unconventional Peptides to HLA-B*58:01

Xuelu Huan[1], Nicole Zhuo[1], Haur Yueh Lee[2], Ee Chee Ren[1, 3*]

[1] Singapore Immunology Network, A*STAR, Singapore 138648;

[2] Allergy Center and Department of Dermatology, Singapore General Hospital, Singapore 169608;

[3] Department of Microbiology & Immunology, National University of Singapore, Singapore 117545.

*Corresponding author:

Ee Chee REN

Singapore Immunology Network, A*STAR;

8A Biomedical Grove, Singapore 138648

Email: ren_ee_chee@immunol.a-star.edu.sg

(a) (b) (c)

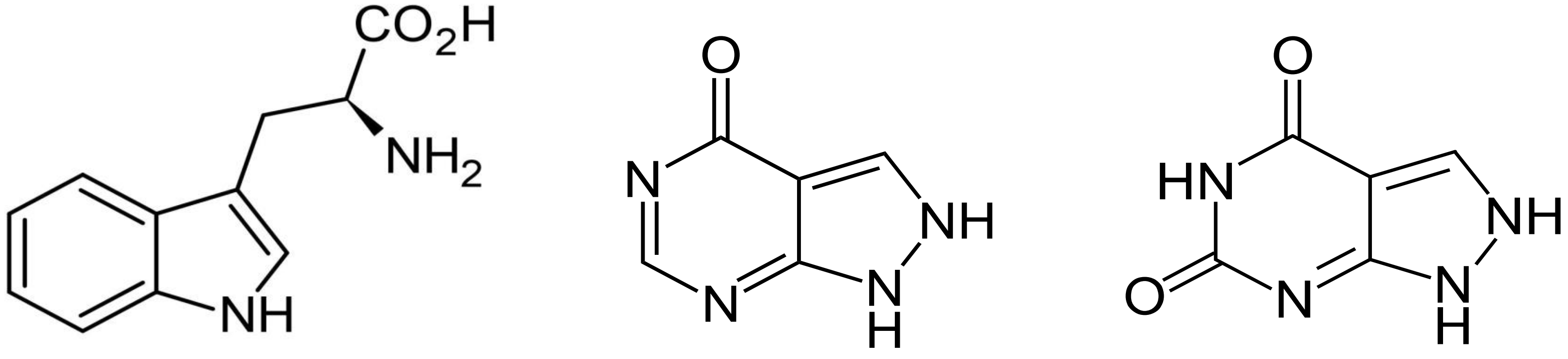

Tryptophan **Allopurinol** **Oxypurinol**

Supplementary Figure 1. Chemical structures of (**a**) Tryptophan, (**b**) Allopurinol, and (**c**) Oxypurinol.

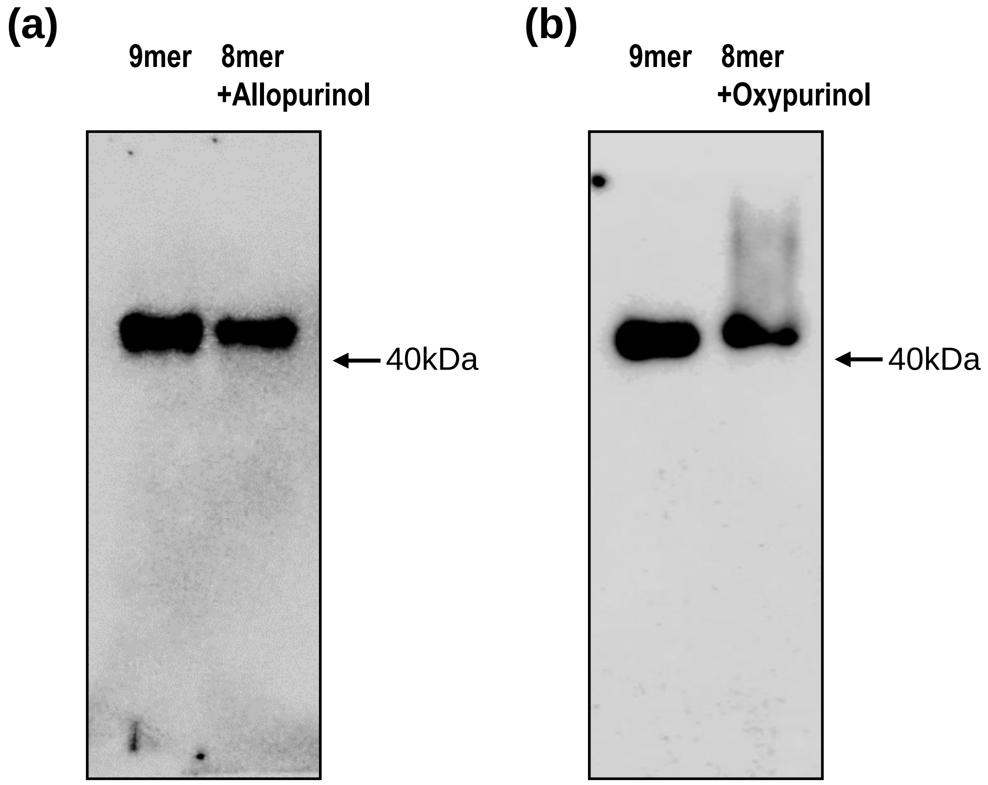

Supplementary Figure 2. The peptide-HLA-B*58:01 complexes purified by size exclusion chromatography were investigated by western blot analysis. (**a**) HLA-B*58:01-KAGQVVTI (8mer) with 10 µg/ml allopurinol. (**b**) HLA-B*58:01- KAGQVVTI (8mer) with 10 µg/ml oxypurinol. Samples of the elution peaks were loaded onto native PAGE gel and transferred onto PVDF blotting membrane. W6/32 mouse monoclonal antibody was used as primary antibody, HRP-conjugated goat anti-mouse antibody was used as secondary antibody. Native HLA-B*58:01-KAGQVVTIW (9mer) complex sample without allopurinol or oxypurinol, was used as a positive control.

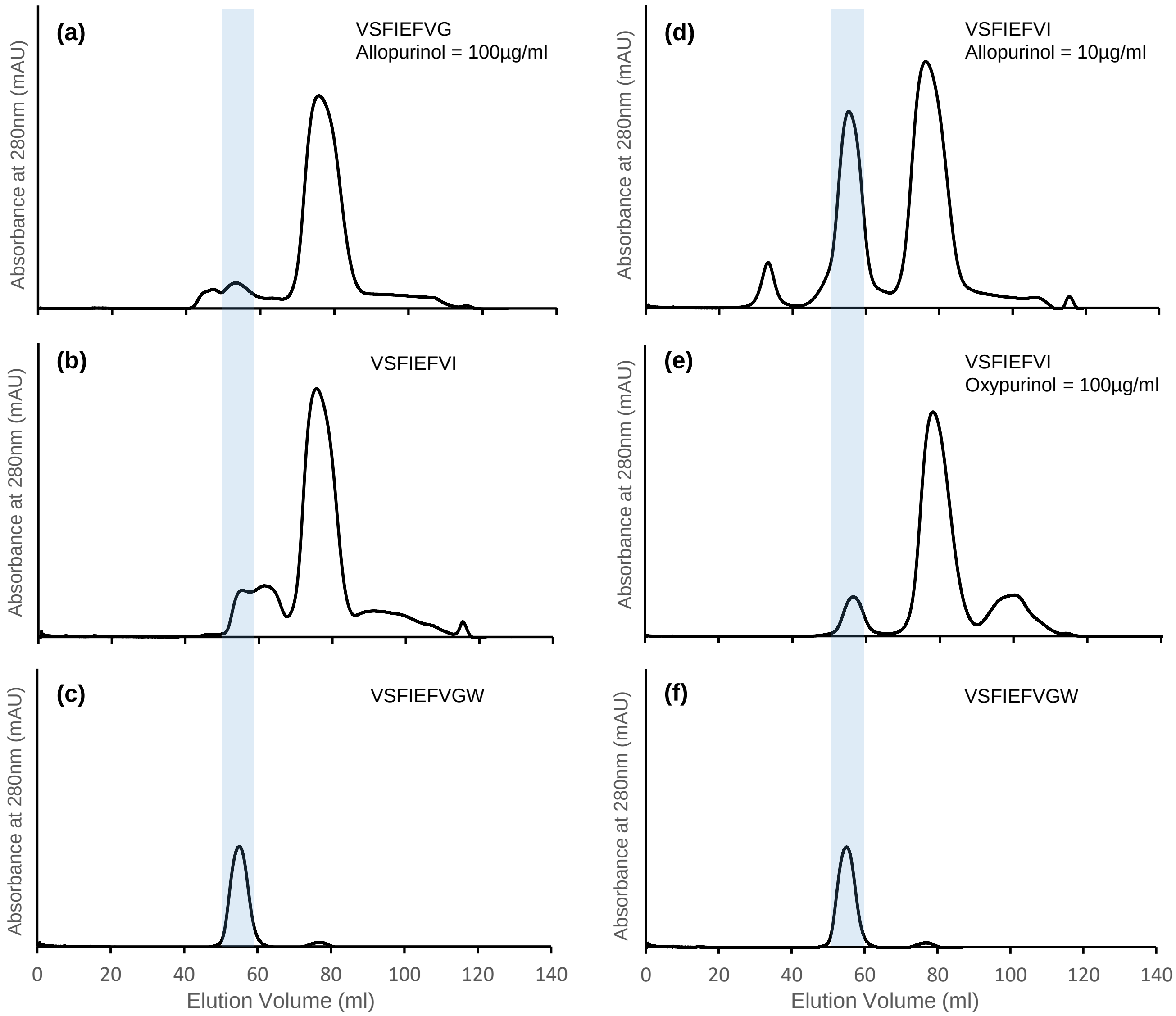

Supplementary Figure 3. FPLC profiles of two different peptide-HLA-B*58:01 complexes with or without the presence of allopurinol or oxypurinol. Size exclusion chromatography with a HiLoad 16/600 Superdex 75 preparatory-grade GF column of HLA-B*58:01 refolded with: (**a**) VSFIEFVG with 100 µg/ml allopurinol. (**b**) VSFIEFVI without allopurinol or oxypurinol. (**d**) VSFIEFVI with 10 µg/ml allopurinol. (**e**) VSFIEFVI with 100 µg/ml oxypurinol. (**c, f**) Native 9mer VSFIEFVGW without allopurinol or oxypurinol, was used as a positive control and elution position marker. The correctly refolded peptide-HLA complex (**c, f**) elutes between 50-60 ml of elution volume and is marked by a vertical light blue bar for easy reference.

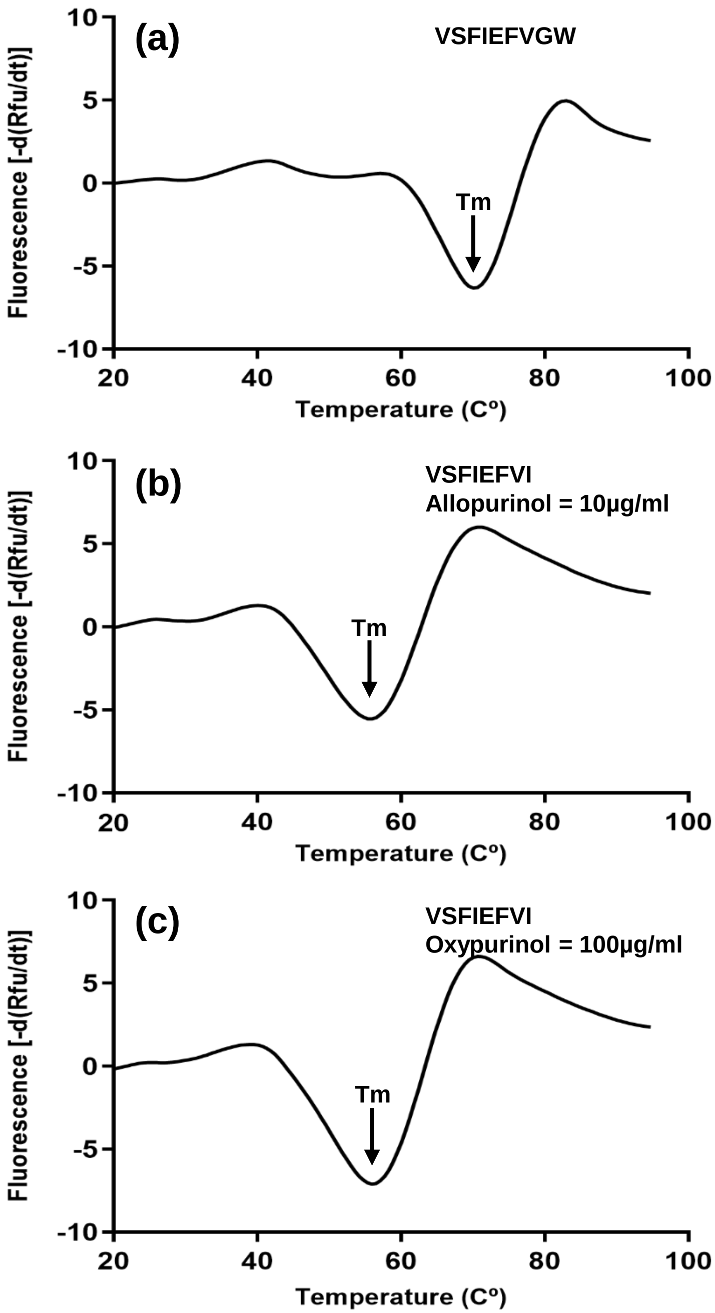

Supplementary Figure 4. Thermal stability of the HLA-B*58:01-peptide complexes. The Tm of (**a**) HLA-B*58:01-VSFIEFVGW, (**b**) HLA-B*58:01- VSFIEFVI with 10 µg/ml allopurinol, and (**c**) HLA-B*58:01- VSFIEFVI with 100 µg/ml oxypurinol was 70 ºC, 57 ºC and 57 ºC, respectively.

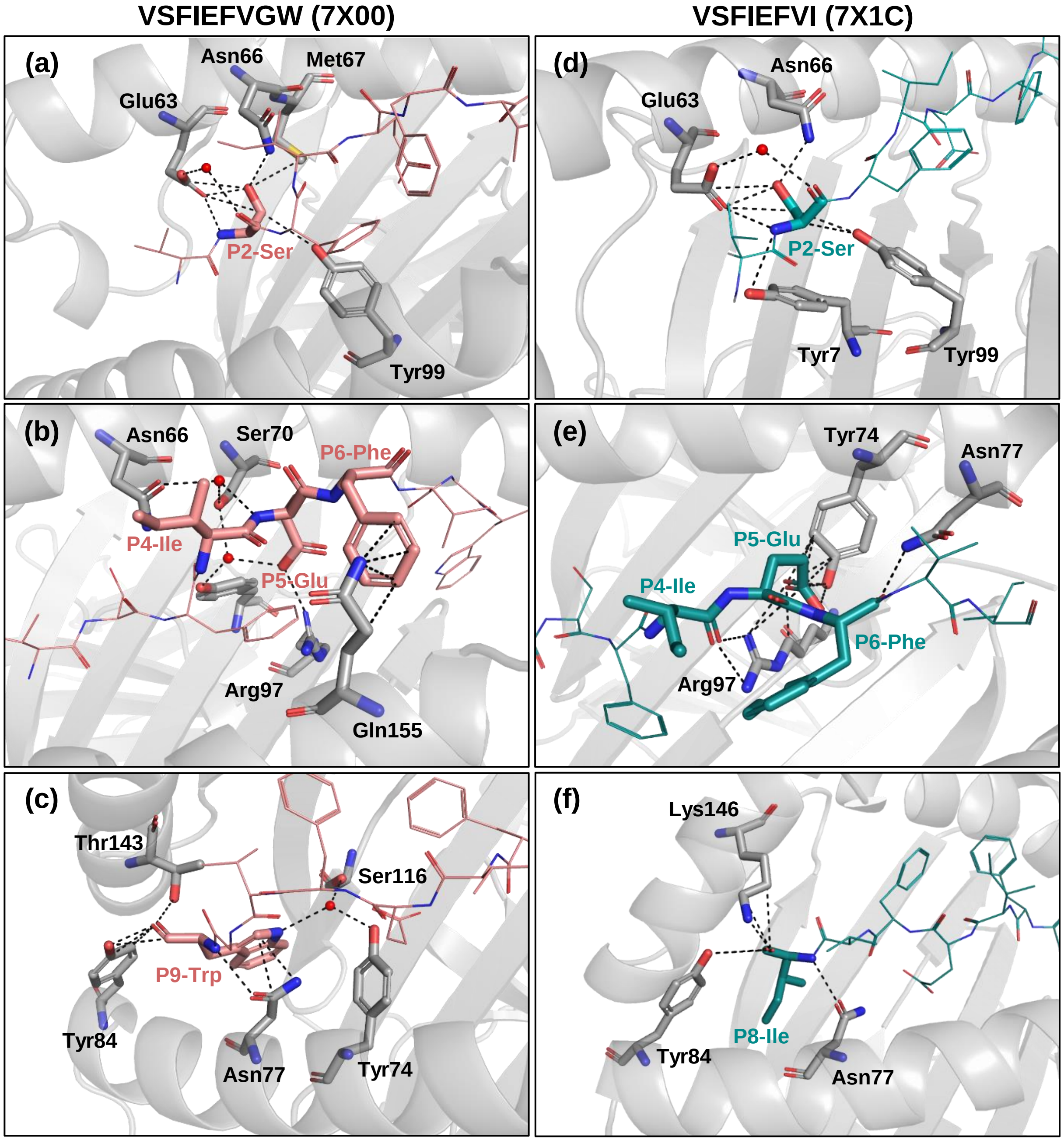

Supplementary Figure 5. Hydrogen bond interactions occurring in allopurinol-peptide-HLA stabilized complex. The main chain of HLA-B*58:01 is depicted schematically in light grey with selected residues shown as stick. Left panel shows interactions for VSFIEFVGW (in light pink, PDB: 7X00), right panel for VSFIEFVI (in teal, PDB: 7X1C). (**a**) shows interactions between P2-Ser of VSFIEFVGW with HLA-B*58:01. (**b**) shows interactions between P4 to P6 residues P4-Ile, P5-Glu and P6-Phe of VSFIEFVGW with HLA-B*58:01. (**c**) shows interactions between P9-Trp of VSFIEFVGW with HLA-B*58:01. Similarly, panel (**d**) shows interactions between P2-Ser of VSFIEFVI with HLA-B*58:01, (**e**) shows interactions between P4 to P6 residues P4-Ile, P5-Glu and P6-Phe of VSFIEFVI with HLA-B*58:01. (**f**) shows interactions between P8-Ile of VSFIEFVI with HLA-B*58:01. Hydrogen bonding is indicated as black dotted line, and peptides are displayed as stick.

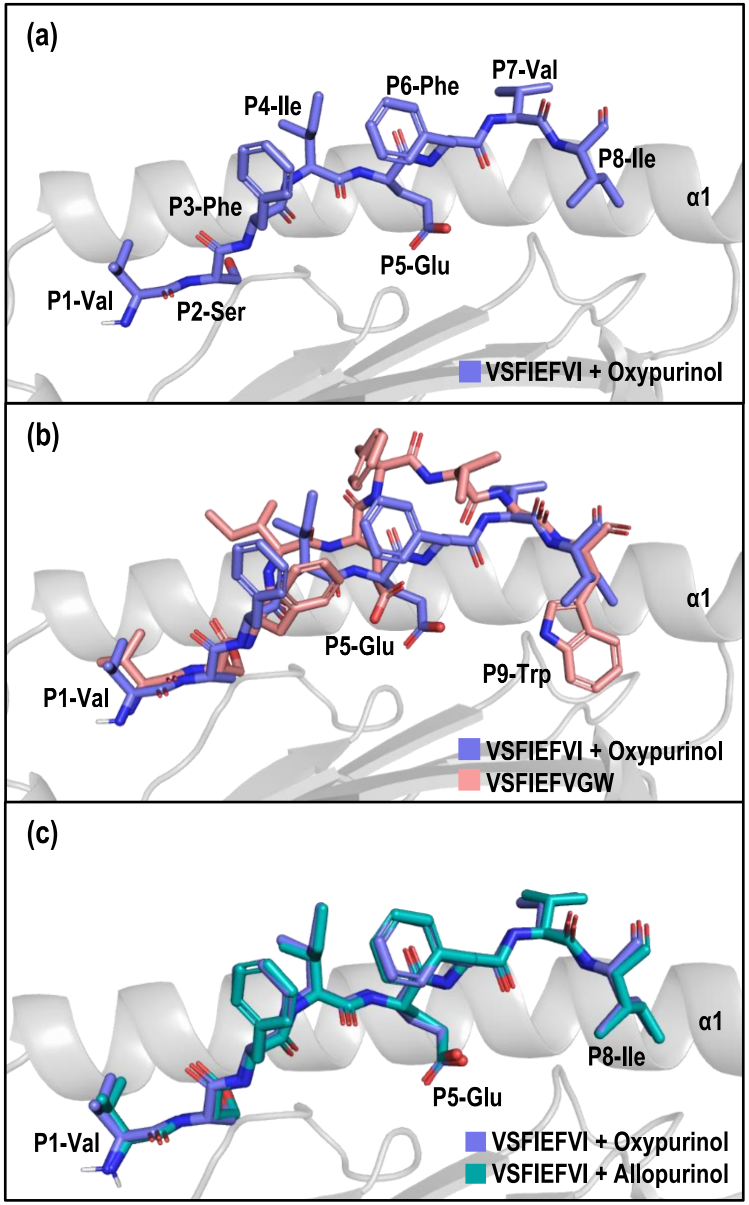

Supplementary Figure 6. Oxypurinol facilitates binding of unconventional 8mer peptide. The main chain of HLA-B*58:01 is depicted schematically in light grey and for clarity only helix-1 (α1) is shown and the peptides are represented in stick. (**a**) 8mer peptide refolded in presence of oxypurinol adopts an extended conformation (purple). (**b**) Overlay of (**a**) and HLA-B*58:01- VSFIEFVGW (light pink) structures clearly illustrates the downward orientation of the middle portion (P4-P6) of the 8mer peptide that enables additional bond interactions with the HLA-B*58:01 molecule. (**c**) Overlay of Allopurinol-VSFIEFVI-B*58:01 (teal) and Oxypurinol-VSFIEFVI-B*58:01 (purple) structures illustrates allopurinol and oxypurinol facilitated VSFIEFVI binding adopt similar configuration.
